# Supplementary figures and images for: Resource diversity and supply drive colonization resistance
Source: PLoS Comput Biol. 2025 Nov 5;21(11):e1013648. doi: 10.1371/journal.pcbi.1013648 (PMC12614786; doi:10.1371/journal.pcbi.1013648)

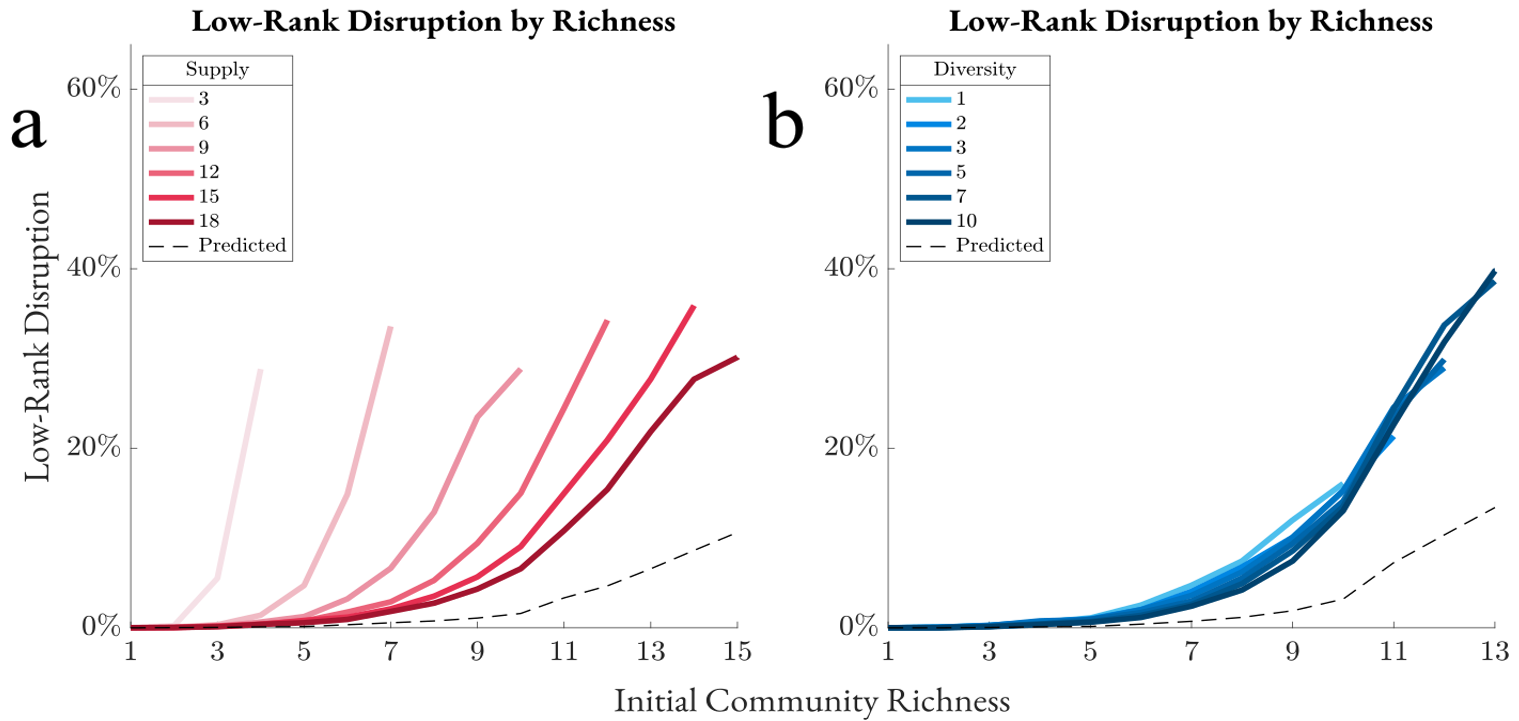

Supplement: S1 Fig — (TIFF) [file pcbi.1013648.s001.tiff]

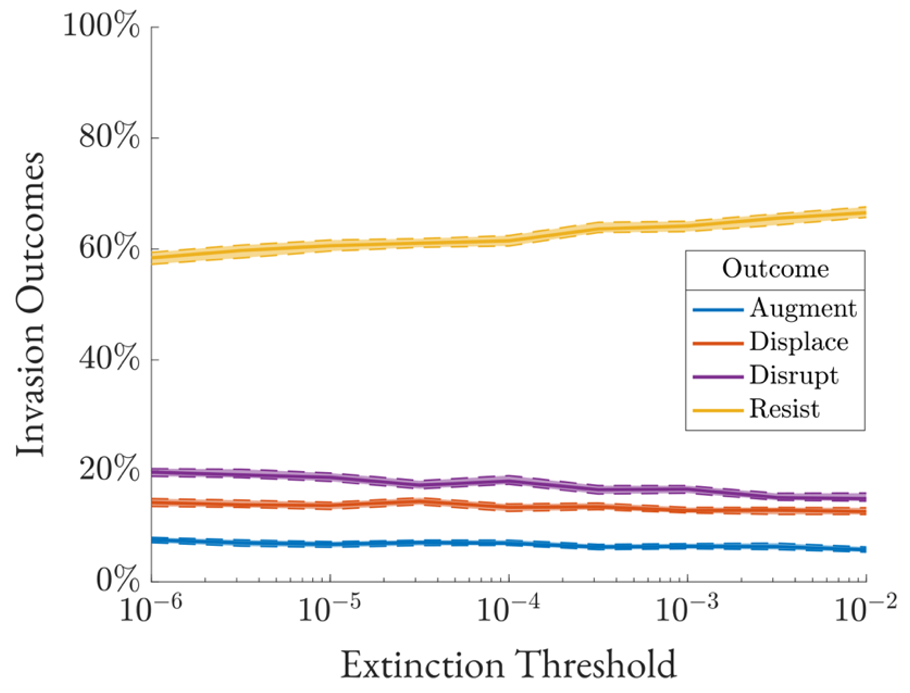

Supplement: S2 Fig — (TIFF) [file pcbi.1013648.s002.tiff]

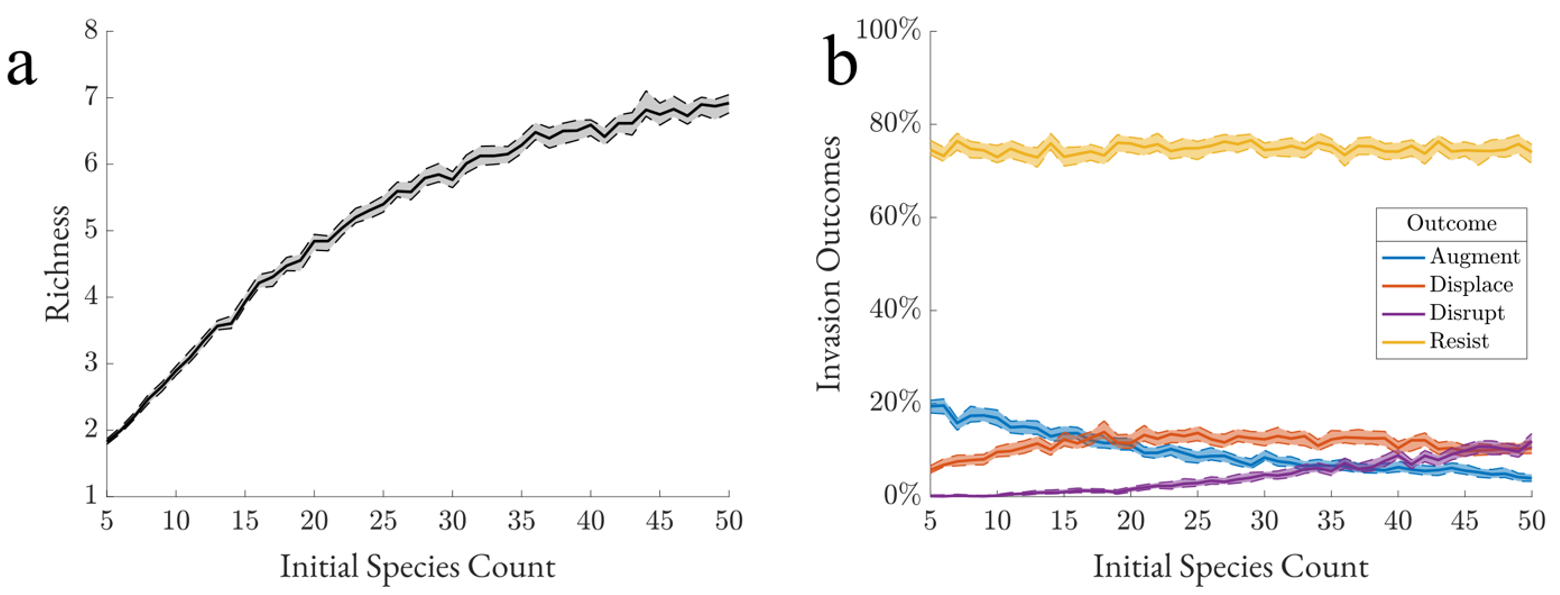

Supplement: S3 Fig — (TIFF) [file pcbi.1013648.s003.tiff]

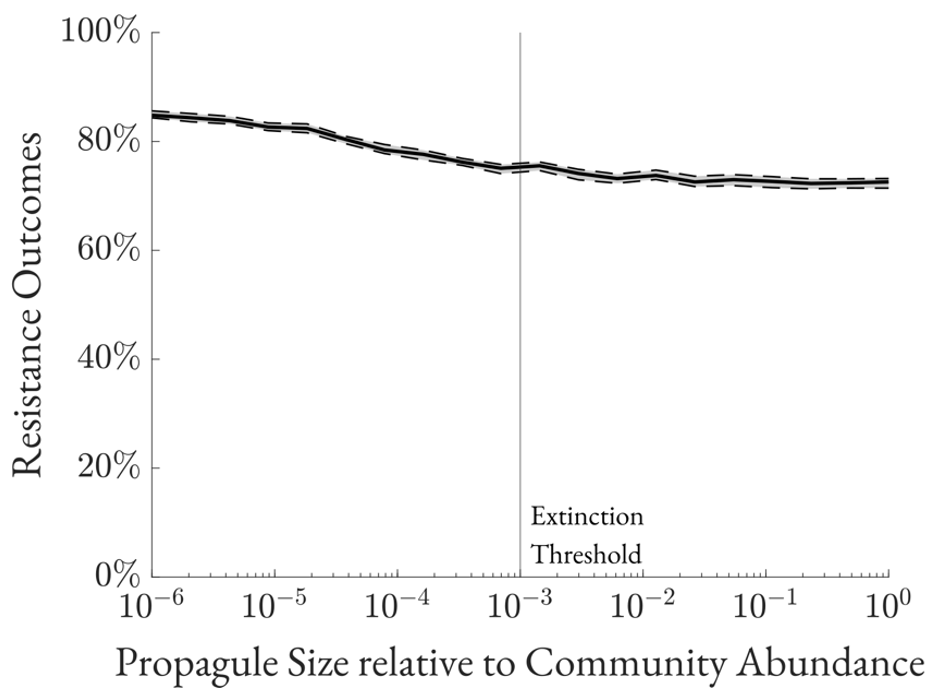

Supplement: S4 Fig — (TIFF) [file pcbi.1013648.s004.tiff]

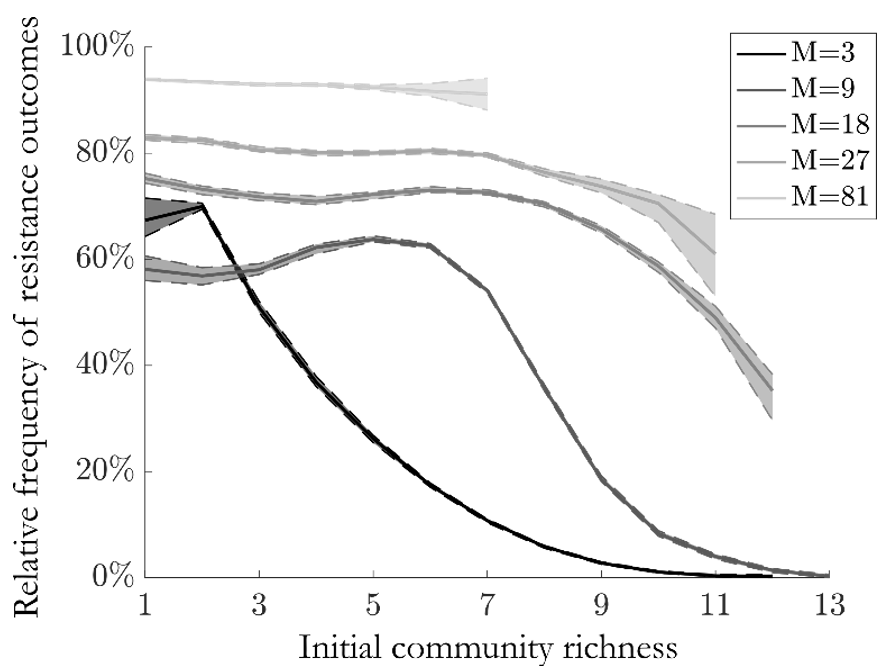

Supplement: S5 Fig — (TIFF) [file pcbi.1013648.s005.tiff]

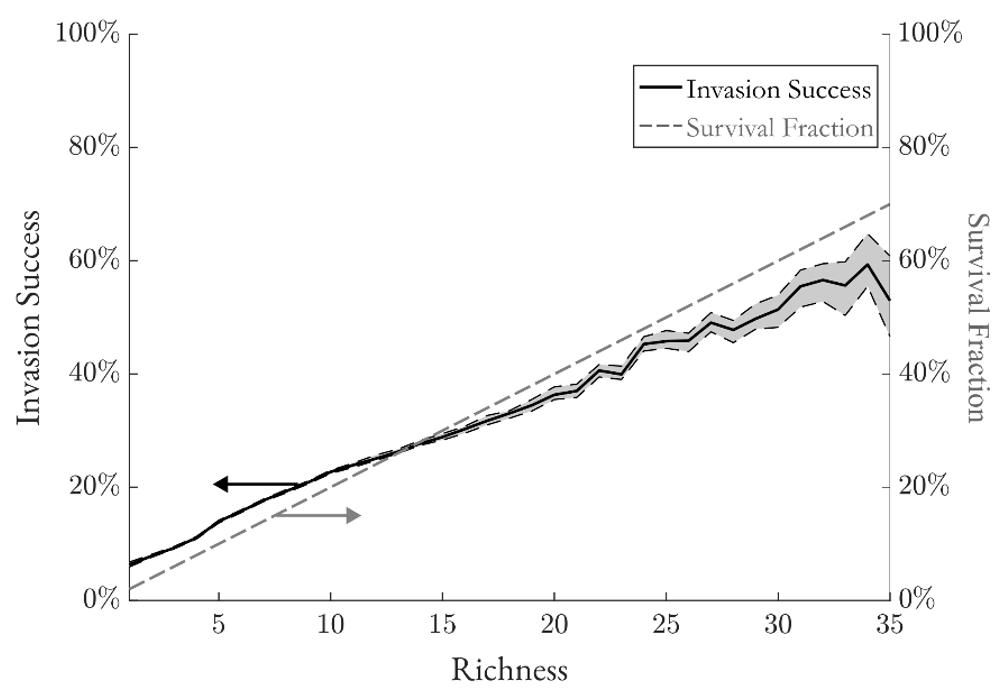

Supplement: S6 Fig — (TIFF) [file pcbi.1013648.s006.tiff]
