# Supplementary material for: Resource diversity and supply drive colonization resistance
Source: PLoS Comput Biol. 2025 Nov 5;21(11):e1013648. doi: 10.1371/journal.pcbi.1013648 (PMC12614786; doi:10.1371/journal.pcbi.1013648)
Supplement: Attachment — Submitted filename: Response to reviewer comments.pdf [file pcbi.1013648.s007.pdf]

Reviewer #1

This manuscript “Resource diversity and supply drive colonization resistance” tackles a fundamental question in microbial ecology: how environmental resource diversity and supply influence colonization resistance in microbial communities. Using a modified version of MacArthur’s consumer-resource modelling (captures the dynamics of species populations and resource concentrations over time) framework coupled with extensive computational invasion simulations, the authors provide insights into how resource factors modulate the relationship between species richness and colonization resistance, as well as the role of cross-feeding dynamics.

This study explores how environmental factors shape the relationship between species richness and colonization resistance. It finds that this relationship is non-monotonic and influenced by both resource supply and diversity. Resistance generally decreases with higher resource supply and often with greater resource diversity—except under resource-limited conditions, where increased diversity surprisingly enhances resistance. Additionally, cross-feeding—which increases resource diversity via metabolic byproducts—can weaken resistance, though this effect vanishes when resources are scarce. Overall, the findings highlight how both external resource inputs and internal metabolic interactions shape a community’s ability to resist invasion. This work advances prior studies focused mainly on species interactions by incorporating explicit resource dynamics, offering valuable perspectives for both natural and engineered microbiomes.

As an experimental biologist, I approach this work from a practical and empirical perspective. While I recognize the value of the modeling framework, my review focuses on the biological relevance of the assumptions and findings, and how well they align with observed microbial behavior and potential for experimental validation and draw parallels within and across microbiomes.

Key contributions
• Non-monotonic richness-resistance relationship: This study reveals that colonization resistance does not simply increase with species richness but follows a complex, resource-dependent pattern, helping to reconcile conflicting empirical observations.
• Cross-feeding effects: Cross-feeding increases resource diversity and can weaken resistance, but this effect disappears under resource limitation, highlighting nuanced ecological interactions relevant for microbiome manipulation.
• Clear communication: The hypotheses, methods, and results are well-articulated, making the study accessible to a broad scientific audience.

We appreciate the clear summary and your insights from the point of view of an experimental biologist.

Strengths
• Advanced modeling approach: The integration of consumer-resource dynamics with cross-

feeding and invasion assays offers a systematic understanding beyond classical ecological models.
• Robust computational design: The authors simulate millions of community replicates with bootstrap confidence intervals, ensuring strong statistical support.
• Relevance: The work addresses the “invasion paradox” by demonstrating how resource supply and diversity condition the effect of richness on invasion resistance.
• Reproducibility: Code availability via GitHub enhances transparency and facilitates future research.

Limitations and suggestions for improvement
Model realism and assumptions
• The model abstracts away spatial heterogeneity, host influences, and evolutionary processes. These simplifications should be explicitly acknowledged, with discussion on how they may affect the generalizability of conclusions.

The model, by design, is simplified to allow us to interpret the findings and draw conclusions. We agree with you that spatial heterogeneity, host influences, and evolutionary processes are important and impactful factors. We have added to discussion the need to study these factors in future investigations.

> “Additionally, spatial structure and heterogeneity, the influence of the host, and evolutionary processes are not accounted for in our current investigation. These are important factors that need to be investigated in future studies.”

• The parameterization of resource types, supply rates, and cross-feeding interactions requires stronger empirical grounding or comparison to real microbial systems.

The implementation of the consumer-resource model used in our manuscript follows the work of Marsland et al, in which two biologically relevant concepts are incorporated in the model assumptions: (1) Energy considerations: each resource is assumed to carry a certain amount of energy, and a balance in energy expenditure is already in place as resources are converted to other resources and taken up by other consumers. (2) Metabolic trade-off: each consumer can utilize some of the resources, but the total uptake across all resources is fixed to capture the trade-off in resource utilization strategy. The model, although clearly simplified, is considered to be a reasonable abstraction of consumer-resource interactions among microbes and has been adopted by several papers before (Estrela et al., eLife, 2021; Diaz-Colunga et al., PNAS, 2022; van den Berg et al. Nature Ecology & Evolution 2022). We have added the following to clarify the rationale behind the use of this model and its parameters.

> “This model is chosen based on the formulation in [38], in which two biologically relevant concepts are incorporated: (1) Energy considerations: each resource is assumed to carry a certain amount of energy, and a balance in energy expenditure is already in place as resources are converted to other resources and taken up by other consumers. (2) Metabolic trade-off: each consumer can utilize some of the resources, but the total uptake across all resources is fixed to capture the trade-off in resource utilization

strategy. The model, although simplified, is considered to be a reasonable abstraction of consumer-resource interactions among microbes and has been used in several previous reports (e.g. [37,39,40]). Arbitrary units are used for resources and population densities in the model, and other parameters such as uptake rates are chosen accordingly in a range that would be consistent with typical microbial growth and uptake rates."

• Empirical validation: This study is purely computational. Incorporating comparisons with experimental or observational data or at least discussing how the predictions align with existing empirical findings would significantly strengthen the manuscript.

We agree with you that empirical validation would significantly strengthen the paper. However, unfortunately the type of empirical evidence required for this validation is not currently available, because of the difficulty/cost of quantitatively profiling all the relevant metabolites and metabolic byproducts in a given community. We hope that posing the idea of resource-driven colonization resistance–computationally discussed in this manuscript–could inspire future empirical investigations when the necessary tools and techniques for such studies become more accessible.

• Parameter sensitivity: Beyond leakage and cross-feeding spread, additional parameters (e.g., resource uptake rates, species pool composition, extinction thresholds) deserve systematic sensitivity analyses to confirm robustness.

Thank you for your suggestion. In fact, we ran many of these sensitivity analyses in the process of preparing the simulations. Many of these parameters represent sums of distributions. For example, total species uptake rate (μ_c) is the sum of the species' uptake rate of each resource. With many such parameters, we found the distribution of elements, i.e. individual resource uptake rates, rather than the total quantity determined species success. To demonstrate this idea, we have included another supplementary figure (Fig S5) which shows how invasion outcome is affected by the number of resources M. When we varied M, we found similar results to when we varied the number of input resources E. Thus, the fraction of input resources to total resources E/M appears to be a greater indicator of community dynamics than simply M.

Species pool composition is determined only by one parameter in our simulations: size (S_0). We provide an in-depth analysis on this parameter in the supplementary figures (Fig S3). As for the metabolic strategies of the initial species pool, we use a randomly generated vector of size M to ensure inclusion of all strategies, generalist and specialist. See Methods for more information on how this vector is generated. We analyzed invasion outcomes under varying extinction thresholds (Fig S2) and found slightly more resistance in communities with higher thresholds, as an invader must reach a higher population threshold to remain. However, this difference is minor and all other invasion outcomes see even smaller changes with respect to the extinction threshold.

Statistics
Unfortunately, the provided information does not sufficiently evaluate whether the statistics performed in the paper are sound and well-written. None of the results appear to directly quote or discuss the statistical methods section of the paper.

We acknowledge this oversight and have added more information about the statistical methods used in this manuscript (see below).

Authors should include:

• Appropriateness of the statistical tests used for the data types and experimental designs

Thank you for the suggestion. In the revised version of the manuscript, under the 'Methods' section, we have added the description of the bootstrapping method that we use to estimate the confidence intervals.

• Adequate description and justification of the statistical models and assumptions

We have added the following to clarify the statistical analyses used in the manuscript.

> "STATISTICAL ANALYSIS
>
> The relative frequency of each invasion outcome is calculated by dividing the number of instances with that invasion outcome to the number of all simulated instances for that condition. To calculate the confidence intervals of the relative frequency of each invasion outcome, we used the bootci bootstrap routine in Matlab. For each data point, 95% confidence intervals are reported, using 1000 bootstrap samples."

• Proper handling of replicates, controls, and data normalisation procedures

We have added more details about the reported data (as 'Relative frequency of invasion outcomes') to clarify that each data point is a frequency obtained from running many samples with randomly selected parameters (typically at least 100,000 per data point). The normalization reflects how often a particular invasion outcome (e.g. resistance) is observed.

> "In this analysis, we quantify the relative frequency of observing different invasion outcomes (Augment, Displace, Disrupt, or Resist) when simulating many instances of invasion with parameters picked randomly from the corresponding distributions (Fig 2 and Table 1)."

• Reporting of effect sizes, confidence intervals, and statistical power

We have taken a conservative approach and used typically more than 100,000 samples for each of the conditions examined. Even though this choice might have resulted in longer-than-necessary computation time, it offers the advantage that the resulting confidence intervals are small and we have high confidence in the reported trends. The sample size is now reported in the caption of each figure.

• Data processing and analysis workflows and significance testing, where appropriate.

To avoid over-cluttering the trends, we did not include the confidence intervals in some of the figures (e.g. Figures 3 and 4 in the original submission; now Figures 4 and 5). We have now included the corresponding figures with confidence intervals to address your concern.

Broader context
The findings contribute meaningfully to ecological theory by elucidating how environmental resource factors mediate colonization resistance and community invasibility. Expanding the discussion to connect with broader invasion ecology literature, empirical work, and potential applications, such as microbiome management strategies, would enhance the manuscript's impact.

Thank you for the suggestion. We have added the following paragraph to the Discussion section.

> "In this study, we classify different resistance regimes of resource input parameter space and show how the richness-resistance relationship is affected in these different regimes. Our work using the consumer-resource model reveals insights into how the environment shapes colonization resistance in microbial communities. Previous studies on invasion ecology often separate two determinants of invasion success: the available environmental niche that would be receptive to the invader and the biotic interactions, including the effect of resident community members, that can facilitate or inhibit the invader. Here we show that in a consumer-resource model that these two factors are tightly linked through the resources present in the environment, the amount and variety of resources supplied in the environment will become the main driver of invasion outcomes. Our findings offer an important insight and a new dimension for microbiome management strategies by controlling the amount and diversity of supplied resources into the environment when possible."

Overall, this manuscript presents an exciting theoretical framework with interesting insights into microbial colonization resistance. Addressing the above points—particularly empirical validation, parallels between this and other studies, expanded sensitivity analyses, and improved clarity—will elevate the work's rigour and relevance, making it suitable for publication.

Reviewer #2

This article investigates how environmental resource diversity and supply influence colonization resistance in microbial communities, utilizing a consumer-resource model and computational invasion simulations. The authors explore the richness-resistance relationship, overall colonization resistance, and cross-feeding dynamics. Key findings include a non-monotonic relationship between species richness and resistance, which is influenced by environmental characteristics. They found that colonization resistance is negatively correlated with both resource supply and diversity, except when resource supply is limited. Additionally, cross-feeding generally weakens colonization resistance by increasing resource diversity, but this trend diminishes under limited resource supply. The study highlights the importance of nutrient blocking and environmental filtering as mechanisms driving increased resistance in resource-limited or species-rich environments.

Major Critiques:
Methods
Numerical analysis: The authors state that the consumer-resource model was encoded in MATLAB and solved using ode45, and that all results are based on these numerical integrations. However, it remains unclear how the simulations map onto each key result (e.g., Figs 3–5). For example, how exactly were resistance proportions computed as a function of supply or diversity? Were species initialized with a fixed or randomized richness for each trial? Was the invader always introduced at the same abundance and time point? A summary table or flowchart outlining which parameters were varied in each experiment, how outcomes were classified, and how these relate to the model equations would greatly improve clarity and reproducibility.

Thank you for the suggestion. We have added clarifying descriptions and an additional figure (now, Figure 2) to describe the entire process of simulating invasion instances and calculating the frequency of each invasion outcome (e.g. resistance).

> “Invasion Assay
>
> To investigate invasion outcomes, we follow a two-step procedure. In the first step (‘assembly’ in Fig 2), a pool of species with randomly selected consumption and conversion profiles are grown together through several passages until a subset of these species reach stable coexistence. We then introduce an invader into these resident communities and examine follow the community to examine if the invader establishes in the community and if all the members of the resident community persist (‘invasion’ in Fig 2). We label the invasion outcomes as one of four possible outcomes (Augment, Displace, Disrupt, or Resist), according to the observed results in each particular instance of the simulation.”

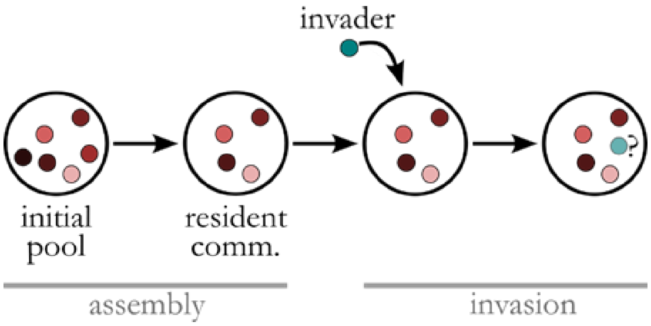

Figure 2: Invasion into communities assembled from randomly interacting species is examined. In the first stage, resident communities are assembled by allowing a pool of randomly interacting resources in the CR model to reach a steady state as the resident community. An invader is then introduced into the community and the community is simulated until it reaches a new steady state. The success/failure of the invader and a possible change in the richness of the resident members is used to categorize the invasion outcomes, as described in Fig 1.

We have also emphasized the procedure again in the Results section for clarification:

> "In this analysis, we quantify the relative frequency of observing different invasion outcomes (Augment, Displace, Disrupt, or Resist) when simulating many instances of invasion with parameters such as consumption rates and conversion rates are picked randomly from the corresponding distributions (Fig 2 and Table 1)."

Microbial consumer-resource model / parameter selection: Authors did not represent what the 18 resources types represent. Are these different carbon sources, or a mix of carbon, nitrogen, and other nutrients? This abstraction limits the biological interpretation of resource diversity and cross-feeding.

Thank you for the clarifying question. In the current implementation of the model and following the formulation by Marsland et al., the 18 possible resources are co-utilized and can be treated for example as different carbon sources. Under this assumption, enough supply of any of these resources can support the growth of a species that can utilize that resource. We have added the following to the Methods section to make this point clear.

> "This model is chosen based on the formulation in [38], in which two biologically relevant concepts are incorporated: (1) Energy considerations: each resource is assumed to carry a certain amount of energy, and a balance in energy expenditure is already in place as resources are converted to other resources and taken up by other consumers. (2) Metabolic trade-off: each consumer can utilize some of the resources, but the total uptake across all resources is fixed to capture the trade-off in resource utilization

> strategy. It should be noted that in this model, different resources are co-utilized and enough supply of any of the resources can support the growth of a species that can utilize that resource."

We agree with you that this is a limitation of the model, as it does not account for irreplaceable resources such as a necessary nitrogen source required in addition to available carbon sources. We have now mentioned this limitation in the Discussion section for more clarity on the scope of our findings.

The concept of "environmental diversity" is central to the manuscript but left undefined in biological terms.

Thank you for the suggestion. We have added the following paragraph to address this point.

> "In this study, we classify different resistance regimes of resource input parameter space and show how the richness-resistance relationship is affected in these different regimes. Our work using the consumer-resource model reveals insights into how the environment shapes colonization resistance in microbial communities. Previous studies on invasion ecology often separate two determinants of invasion success: the available environmental niche that would be receptive to the invader and the biotic interactions, including the effect of resident community members, that can facilitate or inhibit the invader. Here we show that in a consumer-resource model, these two factors are tightly linked through the resources in the environment. This finding in particular highlights that resource supply—defined as the amount of resources supplied by the environment—and resource diversity—defined as the variety of supporting resources for consumers—as major determinants of invasion outcomes. Our findings offer an important insight and a new dimension for microbiome management strategies by controlling the amount and diversity of supplied resources into the environment when possible."

The authors randomly select E resource types from a pool of 18, but the nature of those resources remains unclear. Without examples, it is difficult to interpret whether high diversity refers to nutrient-rich gut environments, diverse carbon substrates. A qualitative mapping or explanatory figure is strongly recommended.

Thank you for your input. We do not specify the exact nature of the resources in the model to preserve generality in applying it to different systems. However, one intuitive context for these resources is diverse carbon substrates, as you mention, where complex carbon sources may be consumed by species A and excreted as new carbon sources which may be consumed by species B. We illustrate this in Figure 1, where 1a shows the species-resource dynamics of the model and 1b shows how we vary the resource input.

Model assumes static resource preferences and a fixed metabolic matrix for all species, which

may not capture the metabolic plasticity and adaptive behaviors observed in real microbial communities.

We agree with you about this limitation. Based on your suggestion and other reviewers' comments, we have included this explicitly as one of the limitations of our study in the Discussion section of the revised manuscript.

> "We recognize that the assumptions made for our model may limit its experimental applicability. One limitation is that the resources in our model are co-utilized and could, for example, represent alternative carbon sources. As such, the current model does not capture situations in which multiple resources are required simultaneously for growth (e.g. a carbon source and a nitrogen source). Additionally, parameterizing cross-feeding dynamics by a single, static matrix does not fully capture the diversity of possibilities such as independent resource requirements (e.g. carbon versus nitrogen sources), preferred utilization of certain resources [46], or metabolic plasticity [47]. We also assume a consistent environmental resource supply and allow communities to reach a steady state before perturbation and invasion occur, but in nature, microbial communities are not necessarily in equilibrium. Moreover, spatial structure and heterogeneity, the influence of the host, and evolutionary processes are not accounted for in our current investigation. These are important factors that need to be investigated in future studies. Despite these limitations, the consumer-resource model is an effective framework for predicting resource-driven niche space. Further investigation is needed to explore how spatiotemporal variation in resource supply and species populations impacts colonization resistance, both within consumer-resource models and in other theoretical frameworks."

The model presumes a constant environmental resource supply and equilibrium before invasion, whereas natural communities often experience fluctuating conditions and may not reach steady state.

We agree with you about these limitations and have explicitly mentioned them now in the revised version (please see the paragraph above). Assuming that the community assembly has reached a steady state before the invader is introduced reflects situations in which the environmental fluctuations are not drastic. Clearly, this assumption is not always applicable. Nevertheless, our previous work suggests that the effect of temporal fluctuations on community composition may not be large (Dedrick, et al., Frontiers in Microbiology 12: 613109, 2021). We thus speculate that our findings could possibly be applied to more general cases, but more rigorous future studies are needed to assert this speculation.

The model does not account for other important ecological interactions such as antimicrobial production, spatial structure, or host factors, which can significantly influence colonization resistance.

Thank you for mentioning these important factors. We have now clearly stated the limitations of the consumer-resource model in the Methods section and the absence of spatial structure and host factors in the Discussion section. We agree with you that these factors can be impactful in determining the outcome of invasion. The focus on consumer-resource interactions in this manuscript is intended to highlight how this particular factor can influence invasion, without discounting other important factors.

Minor Critiques:
Although the manuscript introduces two differential equations at the heart of the consumer-resource model, these equations are never referenced in the text, nor are they numbered. This weakens the link between the mathematical model and the ecological interpretations presented. I strongly recommend the authors number the equations and refer back to them consistently in the Methods, Results, and Discussion when interpreting effects of parameters such as resource supply, cross-feeding, and diversity.

Thank you for the helpful suggestion. We have labeled the equations and updated the manuscript to better integrate the model with the results and discussions, following your recommendation.

Reviewer #3

Understanding the relationship between community diversity / richness and ability to resist invasion is a long-standing challenge in ecology and microbiology. This paper uses a consumer-resource model with cross-feeding to explore how environmental factors—specifically resource diversity and supply—affect colonization resistance in microbial communities. The use of a resource explicit model allows the authors to investigate the richness-resistance relationship and how it depends on resource supply and diversity. The authors argue that there is frequently a non-monotonic richness-resistance relationship that is shaped by environmental conditions. Overall, I found the paper to be interesting, although as discussed below I wouldn't characterize the observed curves as being non-monotonic, as any initial increase in resistance with richness is very modest.

Major points:

Figure 2a: What is "resistance outcomes"? From the text, my guess is that it is the fraction of invasions that are successful? This is very important for readers to understand, and the phrase resistance outcomes is very unclear to me. In any case, I would not describe this curve as being non-monotonic, as any increase is very slight. Instead, I would say that it is flat then decreasing.

We have now labeled the axes as 'relative frequency of resistance outcomes' for more clarity. We have also added the following to the text:

> "In this analysis, we quantify the relative frequency of observing different invasion outcomes (Augment, Displace, Disrupt, or Resist) when simulating many instances of invasion with parameters picked randomly from the corresponding distributions (Fig 2 and Table 1)."

The case of disruptions is very interesting to me. Is this caused by alternative stable states in the original community? It reminds me of the case of "transient invaders" that we characterized experimentally (Amor et al, Science Advances (2020)). In any case, the simplest community dynamics would not be expected to lead to disruptions, so understanding the dynamical origin of this is interesting and important. In the cases of disruption, do you see propagule effects (e.g. outcomes that depend on the initial population size of the invader)?

Disruption outcomes were most prominent in species-rich communities relative to the input resource supply. These outcomes largely resulted in the extinction of only low-rank abundance species, without major changes to the overall community composition (Figure S1). Though we didn't directly analyze alternative stable states within individual communities, these results suggest a core community is assembled around the nutrients present, with periphery or "transient" invaders which may consume remaining scarce nutrients and are vulnerable to perturbation. (Experimental contradiction because empirical communities are generally stable prior to perturbation)

"We note that disruption outcomes in this parameter space occur more frequently within the model than experimentally. One potential reason for this observation is that a community may have alternative steady states, which may be traversed when a "transient" invader sufficiently perturbs the resident populations and/or resource concentrations [43]. Another explanation is that experimental communities are generally more stable prior to perturbation than simulated ones, where extraneous species face less environmental filtering in the idealized model. Our results suggest a core community is assembled around the nutrients present, with such transient invaders surviving on the remaining scarce nutrients which are vulnerable to perturbation."

Propagule size had little effect on the proportion of disruption events, ranging between 10-14% of all invasions. Likewise, we didn't perform extensive analyses into whether community composition was altered differently for varying propagule sizes. Disruption events are not a focal point of this paper, but we plan to address these confounding questions in future works.

"No additional significant propagule effects were found on the remaining invasion outcomes"

We recently found that the probability of a successful invasion (in the absence of priority effects / alternative stable states) was approximately equal to the survival fraction during assembly of the original community (Hu et al, Nature Eco & Evo (2025)). Is this something that is true in your model? (In your case you start with some number S_0 species then achieve some stable richness, so you have the data to calculate this survival fraction)

Thank you for bringing up this interesting result. We define a successful invasion outcome by the presence of the invader in the final community, that is augmentation and displacement outcomes, and find this correlates with the survival fraction of the original community. We have added a section to the discussion which discusses this similarity.

"To further validate that the model dynamics are capable of replicating empirical studies, we analyze the resistance of the community relative to its survival fraction from the initial species pool. A recent study found that invasion success is approximately equal to the survival fraction of a community from its initial species pool, i.e. a community with 50% of the initial species present will admit an invader about 50% of the time. Using a constant initial species pool size, the consumer-resource model produces a similar trend (Fig S6)."

The model makes many particular decisions (see minor points below), and it was difficult for me to always know when each decision is relevant for any given outcome. To some degree this is inevitable, but the problem is compounded the more additions there are to the model. For example, the minimum resource uptake for survival is presumably required for some of the results at low resource supply (which the authors acknowledge), but there were many such questions in my mind (dilution needed, etc).

Minor points:

I didn’t understand how resource preferences were implemented in the model. Is there co-utilization of resources based simply on the c_i,alpha values? Or do species focus on resources based on a resource preference order?

Thank you for pointing this out. We have added a description to clarify that resources are co-utilized based simply on the c_i,alpha values. There is no sequential shift between preferred resources in the model (e.g. no diauxie). Based also on other reviewers’ suggestions, this clarification is now added both to the Methods section and the Discussion section.

Gamma is called the environmental diffusion rate, which strikes me as confusing. Isn’t it just the resource degradation rate?

The terminology was borrowed from the original paper by Marsland et al. that described the model we use (Marsland, 2020), and was intended to represent how some nutrients are dispersed and lost in the environment without being taken up by other community members. We agree that the terminology is a little confusing. In the revised version, we have used ‘dissipation rate’ instead and explained the motivation behind including this term in the model.

“We universally use 18 total resource types from which to choose supplied resource types and to which consumed resources may be converted. E environmentally supplied resources are chosen at random from the M total resource types, and U concentration rate units are randomly allotted to the chosen supplied resource types.” I don’t understand the difference between 18 and M here.

Our apologies for the confusion. The total number of resources, M, is typically set to be 18 in our simulations. We have corrected this issue in the revised version.

I don’t see how the minimum of one unit of resource uptake for zero growth is encoded in the model. It would be nice if the authors would comment on the importance of this assumption, as it is not standard in these resource models (although is indeed frequently observed in the starvation regime).

The mortality rate included in the model means that a certain amount of uptake is required to simply maintain the population at a certain level. This can be interpreted as a balance between growth and death, or alternatively as the maintenance energy for non-growth-related requirements of cells.

Given that the authors have included a resource degradation rate (and species mortality), is it necessary to also assume a dilution step?

We have included a dilution step to more closely emulate a realistic condition, for example, a dilution step in a lab setting during each passage. In our model, the degradation rate, while included, is much smaller in magnitude compared to this dilution. We agree that larger (and linked) resource degradation rates and species mortality rates could simulate a continuous culture situation and eliminate the need for a dilution step. However, in our current model, we have chosen to use smaller, independent rates and use the dilution step as the primary mechanism for population control.
